# Amide Proton Transfer Imaging in Predicting Isocitrate Dehydrogenase 1 Mutation Status of Grade II/III Gliomas Based on Support Vector Machine

**DOI:** 10.3389/fnins.2020.00144

**Published:** 2020-02-21

**Authors:** Yu Han, Wen Wang, Yang Yang, Ying-Zhi Sun, Gang Xiao, Qiang Tian, Jin Zhang, Guang-Bin Cui, Lin-Feng Yan

**Affiliations:** Department of Radiology & Functional and Molecular Imaging Key Lab of Shaanxi Province, Tangdu Hospital, Fourth Military Medical University, Xi’an, China

**Keywords:** glioma, radiomics, isocitrate dehydrogenase 1 mutation, support vector machine, magnetic resonance imaging

## Abstract

**Background:**

To compare the efficacies of univariate and radiomics analyses of amide proton transfer weighted (APT_W_) imaging in predicting isocitrate dehydrogenase 1 (*IDH1*) mutation of grade II/III gliomas.

**Methods:**

Fifty-nine grade II/III glioma patients with known *IDH1* mutation status were prospectively included (*IDH1* wild type, 16; *IDH1* mutation, 43). A total of 1044 quantitative radiomics features were extracted from APT_W_ images. The efficacies of univariate and radiomics analyses in predicting *IDH1* mutation were compared. Feature values were compared between two groups with independent *t-*test and receiver operating characteristic (ROC) analysis was applied to evaluate the predicting efficacy of each feature. Cases were randomly assigned to either the training (*n* = 49) or test cohort (*n* = 10) for the radiomics analysis. Support vector machine with recursive feature elimination (SVM-RFE) was adopted to select the optimal feature subset. The adverse impact of the imbalance dataset in the training cohort was solved by synthetic minority oversampling technique (SMOTE). Subsequently, the performance of SVM model was assessed on both training and test cohort.

**Results:**

As for univariate analysis, 18 features were significantly different between *IDH1* wild-type and mutant groups (*P* < 0.05). Among these parameters, *High Gray Level Run Emphasis All Direction offset 8 SD* achieved the biggest area under the curve (AUC) (0.769) with the accuracy of 0.799. As for radiomics analysis, SVM model was established using 19 features selected with SVM-RFE. The AUC and accuracy for *IDH1* mutation on training set were 0.892 and 0.952, while on the testing set were 0.7 and 0.84, respectively.

**Conclusion:**

Radiomics strategy based on APT image features is potentially useful for preoperative estimating *IDH1* mutation status.

## Introduction

World Health Organization (WHO) grade II/III gliomas include a heterogeneous group of infiltrative neoplasms with astrocytic and oligodendroglia morphology (Louis et al., 2016). Much interest has been focused on histologic class over the past several decades (Daumas-Duport et al., 1988; Fuller and Scheithauer, 2007). However, highly variable clinical behaviors are not adequately predicted based on the histologic phenotype (van den Bent, 2010; Reuss et al., 2015). Therefore, revealing the specific molecular hallmarks has the potential to reduce bias and improve diagnosis as well as prognosis. Notably, in the 2016 WHO classification of central nervous system (CNS) tumors, grade II/III astrocytomas are molecularly divided into *IDH* mutant, *IDH* wild-type, and not otherwise specified categories, emphasizing the diagnostic and prognostic value of *IDH* mutation status in glioma (Louis et al., 2016).

*IDH*s are critical enzymes that catalyze the oxidative decarboxylation of isocitrate to α-ketoglutarate (α-KG) (Yan et al., 2009). *IDH* mutations occur in up to 75% of WHO grade II/III gliomas, but are rarely found in primary glioblastomas (Hartmann et al., 2009; Yang et al., 2012). Patients with *IDH* mutation were more sensitive to chemoradiation therapy and survived longer than *IDH* wild-type ones (Sanson et al., 2009; van den Bent et al., 2010). Moreover, *IDH* mutation would help stratify grade II/III gliomas into subgroups with distinct prognostic characteristics, therapeutic response, and clinical management (Rohle et al., 2013; Olar et al., 2015; Reuss et al., 2015; Jiang T. et al., 2016).

Currently, *IDH* mutation is determined by immunohistochemical staining and DNA sequencing, both are invasive methods with major limitations associated with inherent sampling bias or inability to predict the patient prognosis before surgical resection (Agarwal et al., 2013). As a non-invasive diagnostic tool, magnetic resonance imaging (MRI) technique plays an important role in determining *IDH* mutation. Although promising, the results of most previous experimental studies are conflicting (Arita et al., 2018; Suh et al., 2018), which has hampered consistent clinical application. Notably, identification of 2-hydroxyglutarate (2HG), the metabolite of mutated *IDH*, with MR spectroscopy (MRS) is suggested to be an optimal strategy in identifying *IDH* mutation (Andronesi et al., 2012; Choi et al., 2012; Pope et al., 2012). However, this technique requires a large tumor volume (de la Fuente et al., 2016) and is time-consuming, which limits its application. In addition, partial volume effects between different tumor regions may obscure the identification of 2-HG in smaller regions.

Because widespread disturbances of cellular metabolism occur after *IDH* mutation, including alteration of amino acid concentrations and enzymatic activity (Reitman et al., 2011), and global downregulation of protein expression (Doll et al., 2017). Therefore, more specific imaging modalities are urgently needed to identify *IDH* mutation. Amide proton transfer weighted (APT_W_) imaging is a promising molecular MR imaging technique developed to non-invasively quantify endogenous proteins and peptides (Zhou et al., 2003). For gliomas, APT_W_ imaging was consistently demonstrating potential for grading (Togao et al., 2014), differential diagnosis (Jiang S. et al., 2016; Yu et al., 2017), and treatment response assessment (Sagiyama et al., 2014). Although APT_W_ imaging has been used in a study (Jiang et al., 2017) to predict *IDH* mutation and encouraging results has been obtained, only univariate analysis was focused on histogram and conventional parameters such as the mean, minimal, or maximal values extracted from manually drawn region of interest (ROI). Higher-dimensional quantitative features from APT_W_ images were not fully utilized.

Recently, radiomics analysis has drawn attention (Gillies et al., 2016; Kotrotsou et al., 2016). A large amount of quantitative high-dimensional features can be extracted, processed and analyzed to discover their associations with underlying pathology and genomics. Currently, radiomics approach based on other advanced MRI techniques are promising in predicting glioma genotype (Li et al., 2018; Shofty et al., 2018) and patient survival (Prasanna et al., 2017). However, radiomics analysis based on APT_W_ images in predicting *IDH* mutation has not been reported yet.

Based on these observations, the purpose of this study was to explore whether radiomics analysis of APT_W_ images could acquire a higher efficacy than commonly used univariate analysis in predicting *IDH* mutation of grade II/III gliomas.

## Materials and Methods

### Patient Population

This prospective single institution study has been approved by the Ethics Committee of Tangdu Hospital (TDLL-20151013) and was also registered to ClinicalTrials.gov (NCT03102112).^1^ From June 2016 to October 2017, a total of 429 consecutive patients with suspected gliomas underwent the MRI scanning.

**FIGURE 1:**
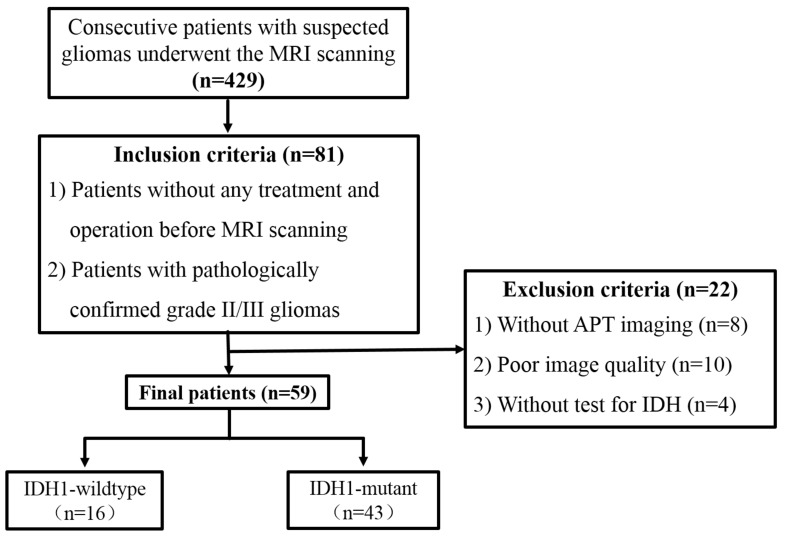
Flow diagram for patient selection.

Inclusion criteria were: (1) receiving no corticosteroid, surgery or any conservative treatment before MRI scan; (2) pathologically confirmed grade II/III gliomas based on the 2016 WHO classification; (3) underwent near total or gross total resection. Eighty-one patients were enrolled in this study. Among them, 22 patients were excluded for the following reasons: (1) without APT_W_ image (*n* = 8); (2) the image quality was unsatisfying with susceptibility or motion artifacts (*n* = 10); (3) without *IDH* information (*n* = 4). Finally, 59 patients were enrolled. The process flow diagram is shown in Figure 1.

### Imaging Data Acquisition

The whole brain MRI examinations were performed on a 3T MRI system (Discovery MR750, General Electric Medical System, Milwaukee, WI, United States) with an eight-channel head coil (GE Medical System). Conventional MRI, contrast-enhanced MRI, and APT_W_ imaging were implemented during the examination.

Conventional MRI scanning included four sequences. (1) axial T_1_-weighted spin-echo image (T_1_WI): repetition time/echo time (TR/TE), 1750 ms/24 ms; matrix size, 256 × 256; field of view (FOV), 24 cm × 24 cm; number of excitation, 1; slice thickness, 5 mm; gap, 1.5 mm. (2) T_2_-weighted spin-echo image (T_2_WI): TR/TE, 4247 ms/93 ms; matrix size, 512 × 512; FOV, 24 cm × 24 cm; number of excitation, 1; slice thickness, 5 mm; gap, 1.5 mm. (3) sagittal T_2_WI: TR/TE, 4338 ms/96 ms; matrix size, 384 × 384; FOV, 24 cm × 24 cm; number of excitation, 2; slice thickness, 5 mm; gap, 1.0 mm. (4) axial fluid-attenuated inversion recovery (FLAIR): TR/TE, 8000 ms/165 ms; matrix size, 256 × 256; FOV, 24 cm × 24 cm; number of excitation, 1; slice thickness, 5 mm; gap, 1.5 mm.

Amide proton transfer weighted imaging was performed prior to the injection of contrast agents. Single-section transverse APT_W_ image at the maximum area of the tumor was obtained with a single-shot echo planar imaging (EPI) sequence using the following parameters: TR/TE, 3000 ms/22.6 ms; matrix size, 128 × 128; FOV, 24 cm × 24 cm; section thickness, 8 mm. Saturation scheme included a total of 4 saturation pulses placed before the EPI readout. The parameters for saturation scheme were as the follows: Fermi-shape saturation pulse width is 500 ms, pulse amplitude 2.0 μT, saturation frequencies include 49 frequency points as well as 3 S_0_ (without saturation pulses). The frequency points were 0, ±25, ±50, ±75, ±100, ±125, ±150, ±175, ±200, ±225, ±250, ±275, ±300, ±325, ±350, ±375, ±400, ±425, ±450, ±475, ±500, ±525, ±550, ±575, ±600 Hz. B_0_ filed map demonstrating the local field shift in Hz was generated from APTw images at the frequency between 275 and −275 Hz.

Finally, contrast-enhanced T_1_WI sequence was acquired in the transverse, sagittal, and coronal planes after intravenous administration of 0.1 mmol/kg gadodiamide (Omniscan; GE Healthcare, Co., Cork, Ireland).

**FIGURE 2:**
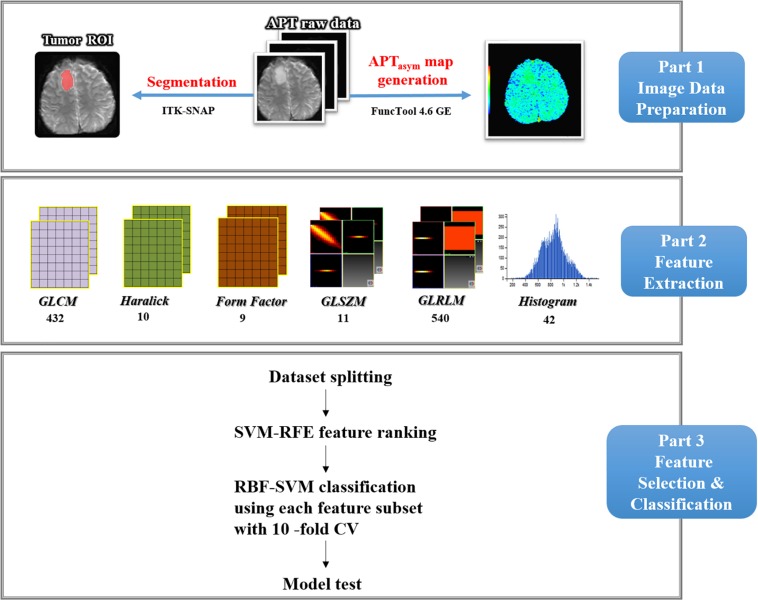
Study flow chart. First, APT_asym_ map was generated from APT raw data and ROI segmentation was done. Second, six types of texture features within ROIs were extracted by using Analysis-Kinetics software, including the histogram, form, GLCM, GLRLM, GLSZM, and Haralick features. At last, automatic glioma *IDH1* mutation classification using RBF-SVM combined with SVM-RFE feature selection and 10-fold cross-validation were carried out. Finally, model was tested by the pre-reserved test data.

After MRI acquisition, all raw data of APT_W_ images were transferred to the workstation (Advantage Workstation 4.6; GE Medical Systems) to generate the B_0_ map and B_0_ corrected magnetization transfer ratio asymmetry (MTR_*asym*_) at 3.5 ppm parametric maps (Part 1 of Figure 2). The APT effect was quantified using MTR_*asym*_ at 3.5 ppm with respect to the water resonance using the following formula:

MTR=asym[S(-3.5ppm)-S(+3.5ppm)]/Swithpixel-by-pixelBcorrection0

### Tumor Segmentation

**FIGURE 3:**
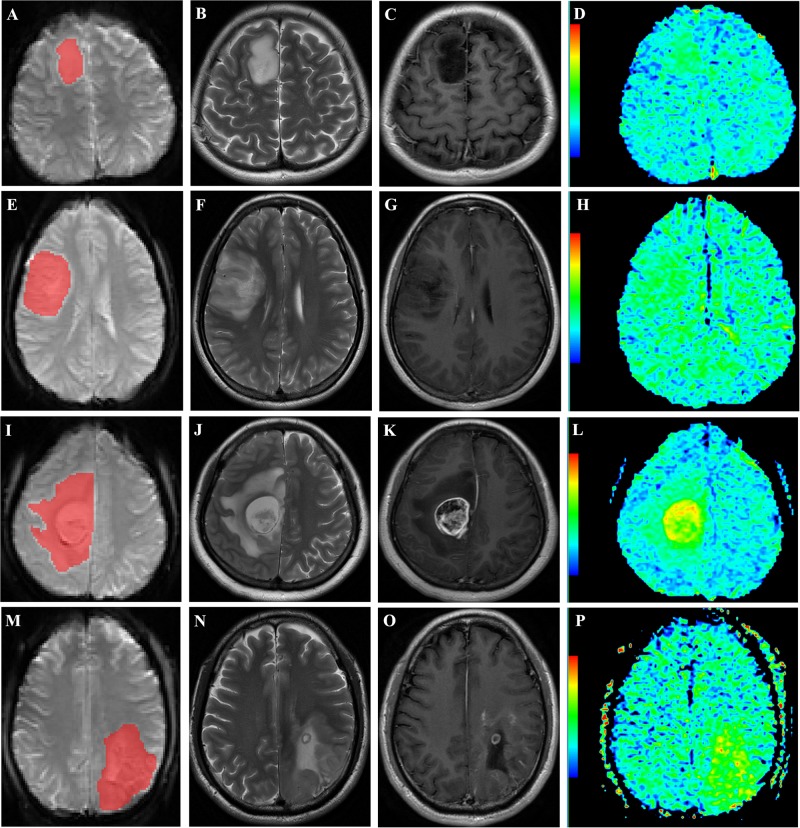
Representative cases. **(A–D)**: a 36-year-old woman with WHO grade II diffuse astrocytoma with *IDH1* mutation in the right frontal lobe. ROI selection is based on S_0_ map of APT raw data **(A)**. The lesion shows hyperintensity on axial T_2_WI **(B)** and no enhancement on postcontrast T_1_WI **(C)**. The APT_W_ image **(D)** exhibits increased signal intensity in the lesion. **E–H**: a 31-year-old woman with WHO grade II diffuse astrocytoma with *IDH1* wild type in the right frontal lobe. ROI selection is based on S_0_ map of APT raw data **(E)**. The lesion shows heterogeneous hyperintensity on axial T_2_WI **(F)** and no enhancement on postcontrast T_1_WI **(G)**. The APT_W_ image **(H)** exhibits increased signal intensity in the lesion. **I–L**: a 31-year-old woman with anaplastic astrocytoma with *IDH1* mutation in the right frontal lobe. ROI selection is based on S_0_ map of APT raw data **(I)**. The tumor and peritumoral edema shows hyperintensity on axial T_2_WI **(J)** and heterogeneous enhancement of tumor on postcontrast T_1_WI **(K)**. The APTw image **(L)** exhibits increased signal intensity in the tumor and peritumoral edema. **(M–P)**: a 45-year-old man with anaplastic oligodendroglioma with *IDH1* wild type in the left parietal lobe. ROI selection is based on S_0_ map of APT raw data **(M)**. The tumor shows heterogeneous hyperintensity on axial T_2_WI **(N)** and ring-like and strip-like enhancement within tumor on postcontrast T1WI **(O)**. The APTw image **(P)** exhibits increased signal intensity in the tumor.

All images were anonymized and stored in DICOM format. Two experienced neuroradiologists (L-FY and GX who have 7 and 5 years of experience, respectively, in neuro-oncology imaging) reviewed the conventional plain and contrast-enhanced MR images carefully to determine the margin of tumor. The APT raw data were imported into the ITK-SNAP software (version 3.6.0)^2^ and the S_0_ map of APT raw data were identified. The contour line of the ROI was drawn manually based on S_0_ map while attempting to maintain an approximate distance of 2–3 mm from the tumor margin to minimize the partial volume effect. As shown in Figure 3, two-dimensional ROI including tumor, possible edema, cystic degeneration and necrosis was acquired for each patient.

### Feature Extraction and Robustness Analysis

As depicted in Part 2 of Figure 2, using a non-commercial Analysis-Kit software (GE Healthcare, China), the method of quantitative feature extraction was conducted on MTR_*asym*_ (3.5 ppm) parametric maps. Forty-two first-order histogram, 983 s-order texture [432 gray level co-occurrence matrix (GLCM), 540 gray level run length matrix (GLRLM), and 11 gray level size zone matrix (GLSZM)], 9 form and 10 Haralick features were extracted from MTR_asym_ (3.5 ppm) parametric maps. Thus, a total of 1044 quantitative features were obtained from the original images. Details regarding the quantitative features extracted in this study are presented in Supplementary Appendix S1.

As all the extracted data depend on the ROIs delineated by radiologists, the robustness of all the features was evaluated by both test-retest analysis and inter-rater analysis. For test-retest analysis, ROIs for each patient were segmented twice by one rater based on 20 randomly selected patients. Another 20 randomly selected patients were independently segmented by two raters for interrater analysis. The features extracted from these ROIs were assessed using the intraclass correlation coefficient (ICC).

### Machine Learning Classification

As shown in Part 3 of Figure 2, the classification procedure included data grouping, data augmentation, feature selection, model building and model testing. First, the majority of patients’ data from each group were randomly selected to train the model, i.e., 11 *IDH1* wild type and 38 *IDH1* mutation. To solve the potential impact of the imbalance dataset in the training cohort, a synthetic minority oversampling technique (SMOTE) was applied to solve this issue and its merit has also been confirmed in our previous study (Zhang et al., 2017). In this study, the number of *IDH1* wild-type patients in training data were augmented to that of *IDH1* mutation, i.e., 0.38. The synthetic cases would have features with values similar to the existing cases and were not merely replications, thus improving the representation of the minority group in the dataset. The data of five patients in each group were left out for testing without applying SMOTE.

Highly correlated or less effective features may lead to the overfitting issue as well as increase the computing burden. Thus, SVM-based recursive feature elimination (SVM-RFE) algorithm was applied to select the most effective features in the training set to prevent overfitting and improve model generalization. It is able to rank the features according to their weight during *N* times iterations (*N* is the total number of extracted features). At each iteration, the feature with the minimal weight was eliminated, leading the feature ranking from the most to the least important one. Then, *N* feature subsets were established by selecting the first n features from the ranked sequence (1 ≤ *n* ≤ *N*). To compare the performance of different feature subsets, each subset was input into SVM with radial basis function (RBF) kernel and tested with 10-fold cross-validation.

All the classifications were performed using Waikato Environment for Knowledge Analysis (WEKA, version 3.8.2). SVM constructs a hyperplane that provides the optimal separation boundary to maximize the separation of the objects in a high-dimensional space, and this approach is widely used because of its stability and favorable performance. In this feature space, a decision surface is created with different subspaces. Each subspace corresponds to one class of training data. Once the training is completed, the test data are mapped to the feature space. A class is then assigned to those data depending on which subspace they are mapped to. Before that, each feature was normalized into the range from 0 to 1. A RBF kernel maps the original data with the kernel function as K(x) = exp(gx−t^2^), where x and t are two feature vectors, and gamma (g) controls the shape of the decision hyperplane. Loss function assesses the degree of inconsistency between the predicted and real values. The parameter g in the kernel function and epsilon in loss function were set to 1/3 (default value, 1/max_index) and 0.1, respectively. Probability estimation and shrinking heuristics were applied. The optimal feature subset would be got according to the classification performance. Then, the test data were put into the model built by using the optimal feature subset to test the performance of the final model.

The classification accuracy and AUC were measured in both training and test cohorts to evaluate the predictive efficiency of the radiomics model.

### Histopathological Evaluation

Resected tumor tissues were processed using standard clinical techniques. *IDH1* R132H analysis was confirmed by immunohistochemistry and DNA sequencing as previously described (Yip et al., 2012; Agarwal et al., 2013). Paraffin sections of the intracranial tumor specimens were stained with *IDH1* R132H mutation-specific antibodies (1:50; H09 clone, Dianova). The *IDH* forward primer (5′-ACC AAA TGG CAC CAT ACG A-3′) and reverse primer (5′-GCA AAATCA CAT TAT TGC CAA C-3′) were designed to amplify exon 4 (codon R132) of the *IDH* gene.

### Statistical Analysis

All statistical analyses were performed by using SPSS 20.0 software (IBM Corp, Chicago, IL, United States), WEKA software (WEKA version 3.8.2), and R software (version 3.3.2). All the extracted features in our study were assessed using ICC for both test-retest and inter-rater analyses. According to a previous study (Gevaert et al., 2014), features with ICC ≥0.6 were considered as robust against intra- and inter-rater uncertainties. The normal distribution of data was investigated with Kolmogorov-Smirnov (K-S) test. The between-group comparisons of quantitative data (age and quantitative features), categorical data (gender, cortical involvement, midline cross and location) were analyzed using independent sample *t*-test and chi-square test. Receiver operating characteristic (ROC) curve analysis was performed to determine the performance of single feature or radiomics model, and accuracy and area under the curve (AUC) were obtained. *P* < 0.05 indicated a significant difference.

## Results

### Patients Characteristics and Feature Robustness

Among 59 patients enrolled in this study, 16 were *IDH1* wild-type (male, 11; female, 5; age range, 3–76 years), and the remained 43 were *IDH1* mutant (male, 25; female, 18; age range, 17–67 years). Age and radiomics features were normally distributed. Statistical results were summarized in Table 1. There were no significant differences in age, gender, midline cross and multi-lobe involvement between the two groups (*P* > 0.05), whereas *IDH1* mutant gliomas were mostly located in the frontal lobe and involving the cortex (*P* < 0.05).

**TABLE 1:** Baseline demographics and clinical characteristics of patients.

	***IDH*-mutant**	***IDH* wild-type**	***P*-value**
Patients (*N*/%)	72.9% (43/59)	27.1% (16/59)	NA
Age (mean ± SD)	44.32 ± 10.68	40.18 ± 19.17	0.298
Gender (*N*/%)			0.333
Male	58.1% (25/43)	68.8% (11/16)	
Female	41.9% (18/43)	31.2% (5/16)	
Cortical involvement (*N*/%)	93.0% (40/43)	56.2% (9/16)	0.002
Cross the midline (*N*/%)	16.3% (7/43)	0 (0/16)	0.173
More than two lobes involved (*N*/%)	37.2% (16/43)	56.2% (9/16)	0.241
Location (*N*/%)			
Frontal lobe	76.7% (33/43)	43.8% (7/16)	0.027
Parietal lobe	4.7% (2/43)	12.5% (2/16)	0.295
Temporal lobe	9.3% (4/43)	18.7% (3/16)	0.375
Other locations	9.3% (4/43)	25% (4/16)	0.194
Histologic subtype (*N*/%)	Diffuse astrocytoma	Anaplastic astrocytoma	
	54% (20/37)	63.6% (14/22)	
	Oligodendroglioma	Anaplastic oligodendroglioma	NA
	46% (17/37)	36.4% (8/22)	

Among 1044 radiomics features, the ICCs of 1038 features for both test-retest analysis (0.823-1) and interrater analysis (0.712-1) were greater than 0.6, thus were robust for further analysis.

### Univariate Analyses for IDH1 Mutation Prediction

Significant differences were observed in 18 out of 1038 extracted quantitative features between the *IDH1* wild-type and mutant groups, including 8 first-order histograms, 6 GLCM and 4 GLRLM. The accuracies and AUC of these features were shown in Table 2. Among 18 features, *High Gray Level Run Emphasis All Direction offset 8 SD* achieved the highest AUC [0.769, 95% confidence interval (CI) 0.641–0.869] with the accuracy of 0.799.

**TABLE 2:** Diagnostic performance of univariate analyses in predicting *IDH1* mutation.

**Parameter**	**Accuracy (%)**	**Sensitivity (%)**	**Specificity (%)**	**AUC (95% CI)**	***P*-value**
**Histogram**					
H1	74.5	76.7	68.7	0.702 (0.569−0.814)	0.015
H2	74.6	76.8	68.8	0.702 (0.586−0.827)	0.019
H3	74.6	74.4	75.0	0.734 (0.603−0.841)	0.003
H4	74.6	76.7	68.8	0.719 (0.587−0.829)	0.007
H5	74.6	72.1	81.2	0.734 (0.603−0.841)	0.004
H6	76.2	76.7	75.0	0.725 (0.594−0.833)	0.006
H7	74.5	76.7	68.7	0.711 (0.578−0.821)	0.011
H8	71.2	69.8	75.0	0.695 (0.561–0.808)	0.003
**GLCM**					
G1	59.3	51.2	81.3	0.696 (0.563−0.809)	0.012
G2	62.7	55.8	81.3	0.664 (0.529−0.782)	0.034
G3	69.1	69.1	68.8	0.710 (0.576−0.822)	0.006
G4	77.6	90.2	43.8	0.691 (0.555−0.807)	0.018
G5	71.3	72.5	54.6	0.687 (0.567−0.789)	0.043
G6	69.0	69.1	68.8	0.710 (0.576−0.822)	0.006
**GLRLM**					
R1	66.1	60.5	81.2	0.670 (0.535−0.787)	0.033
R2	66.1	65.1	68.7	0.667 (0.532−0.784)	0.033
R3	67.8	67.4	68.7	0.712 (0.580−0.823)	0.005
R4	79.9	88.7	56.3	0.769 (0.641−0.869)	<0.001

*H1, RMS; H2, percentile 70; H3, percentile 75; H4, percentile 80; H5, percentile 85; H6, percentile 90; H7, percentile 95; H8, quantile 0.975; G1, correlation all direction offset 2 SD; G2, correlation angle 45 offset 4; G3, cluster shade angle 45 offset 7; G4, cluster prominence angle 45 offset 7; G5, cluster shade all direction offset 8 SD; G6, inverse difference moment angle 135 offset 6; R1, long run high gray level emphasis angle 135 offset 1; R2, short run emphasis all direction offset6; R3, long run emphasis all direction offset 6; R4, high gray level run emphasis all direction offset 8 SD.*

### Machine-Learning for Predicting IDH1 Mutation

**FIGURE 4:**
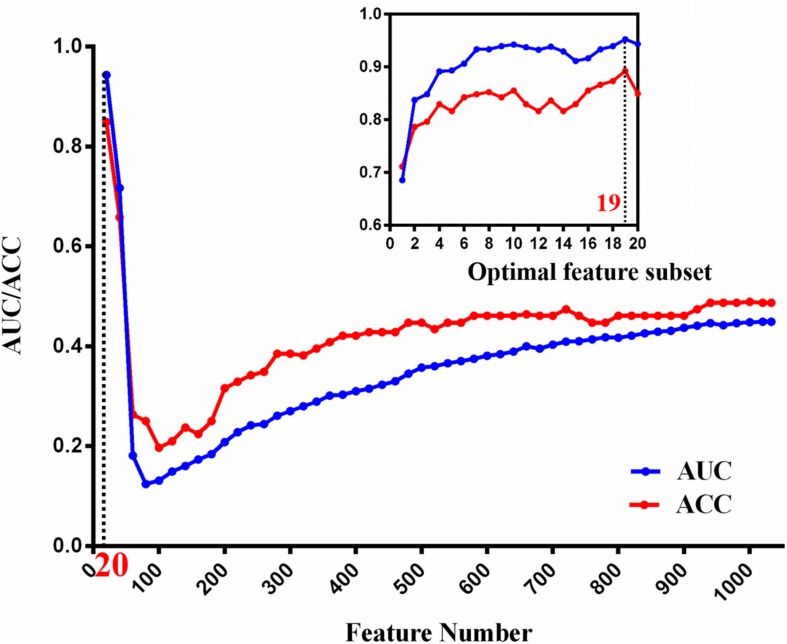
The tendency of classification AUC and ACC value during optimal attribute determination of machine-learning model. The horizontal axis is the attribute number and the vertical axis is the AUC/ACC value. The local classification performance at peak point is magnified to view on the top right corner.

Based on the *IDH1* mutation status, 1038 robust features extracted from APT_W_ images were employed to construct the machine-learning model. Nineteen features were selected to be the optimal feature subset for *IDH1* mutation prediction using SVM-RFE. Figure 4 illustrates the feature selection process. With the participation of new twenty ranked features sequentially, the overall tendencies of classification accuracy first declined and gradually increased. The peak of curve was achieved by using the top-20 feature subset. For the top-20 features, classification accuracy was evaluated again with inclusion of each ranked feature one by one. The best performance was achieved by using the top-19 feature subset, which was determined as the optimal feature subset.

Afterward, the independent test data were used to test the final solution in order to confirm the actual classification power. The test accuracy and AUC of the model were 0.70 and 0.84, respectively.

### Optimal Feature Subset Analysis

**FIGURE 5:**
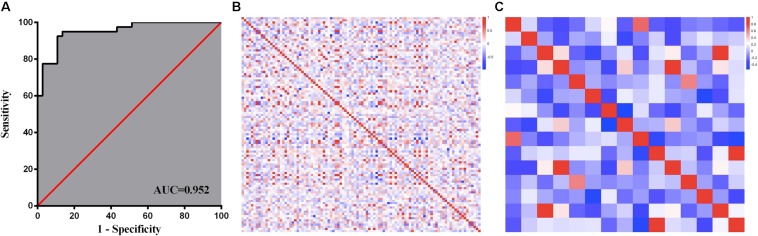
Feature selection and analysis. ROC curve of machine-learning model **(A)**; Heat maps of feature correlation analysis before **(B)** and after **(C)** feature selection.

As described above, 19 out of the 1038 features were selected to be the optimal feature subset to establish *IDH1* mutation prediction model. It can be observed that GLCM features (*n* = 13) accounted for a high proportion, and the rest (*n* = 6) were GLRLM features. Detailed descriptions of the 19 features are listed in Table 3. The ROC and the heat maps of feature correlations before and after feature selection were shown in Figure 5. The machine-learning model achieved better discriminative ability to predict *IDH1* mutation, with the ACC and AUC of 0.892 and 0.952, respectively (Figure 5A). As further demonstrated in Figure 5B, before feature selection, there was mass redundant information among the 1038 features, which appeared as the high correlation coefficients ranging from −1 (blue) to 1 (red). After feature selection, 19 optimal features had a relatively low correlation and high effectiveness, as shown in Figure 5C.

**TABLE 3:** The optimal radiomic features selected by the SVM-RFE method.

**Gray level co-occurrence matrix (GLCM) (*n* = 13)**	**Gray level run-length matrix (GLRLM) (*n* = 6)**
Correlation all direction offset4 SD; GLCM Entropy angle45 offset2; Inertia all direction offset3; Haralick Correlation angle45 offset1; Inverse difference moment angle0 offset5; Inertia angle0 offset2; Cluster prominence all direction offset1 SD; Inverse difference moment angle135 offset1; GLCM Energy angle135 offset3; Correlation angle0 offset1; Inverse difference moment angle135 offset9; Inverse difference moment all direction offset7 SD; Correlation angle135 offset5; Cluster prominence angle135 offset2	Run length non-uniformity angle0 offset1; Low gray level run emphasis angle90 offset3; Long run low gray level emphasis angle45 offset6; Long run low gray level emphasis all direction offset5; Run length non-uniformity all direction offset5; High gray level run emphasis angle0 offset8

**FIGURE 6:**
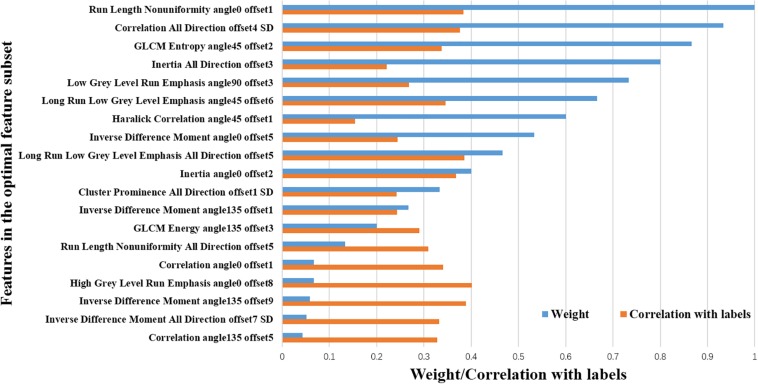
Analysis of the optimal feature subset of machine-learning model. The blue bars represent the contribution weights of the optimal feature subset of machine-learning model. The orange bars represent the correlation coefficient for features and the classification class.

The contributing weight and correlation coefficient between each feature and the classification class of the optimal feature subset were shown in Figure 6. It is obvious that the corresponding trend of the blue and orange bars of each feature does not match, which indicates that the performance of an individual feature does not determine its contribution to the optimal feature subset. That is to say the feature subset does not necessarily require that each feature to be very powerful, but the complementarity between features can help the subset achieve the best results. Therefore, to simply combine several individual parameters with higher classification performance may not achieve enough good results.

## Discussion

In this study, the histogram and high-order features were extracted from APT_W_ images. Both univariate and radiomics analyses were performed to compare the efficacies in predicting *IDH1* mutation. Our SVM model achieved an AUC of 0.952 and 0.84 in the training set and test set, respectively. Notably, the efficacy achieved by SVM model was superior to that of univariate analysis.

Jiang et al. (2017) first confirmed the ability of APT_W_ images in identifying the *IDH1* mutation status. Based on their study, *IDH* wild-type gliomas were associated with relatively high APT_W_ signal intensities as compared with *IDH*-mutant ones. Similarly, our univariate analysis revealed that 8 out of 18 determinant variables were histogram features. Among these histogram features, the higher AUCs (0.734) were achieved by using the 75th and 85th percentile APT_W_ values. However, different from their study, the best diagnostic performance was achieved by using *High Gray Level Run Emphasis All Direction offset 8 SD* with AUC of 0.769. Furthermore, the highest AUC of 0.952 was achieved by using radiomics model. The following factors may contribute to the differences: (1) different study population; only WHO grade II gliomas with relatively small sample size were enrolled in their study. In light of the 2016 WHO classification, the anaplasia and mitotic activity in WHO grade II from III gliomas may result in inter-observer variability. Furthermore, clinical outcome differences of grade II/III gliomas rely far more on molecular subtypes than on grading, suggesting that molecular parameters may in fact be a better tool in identifying subgroups with distinct prognosis. Consequently, in accordance with cancer genome archive (TCGA), we merged WHO grade II/III gliomas as “lower grade glioma”; (2) different ROI strategy; in their study, both whole tumor histogram-based and multi-ROI-based analyses were adopted. The best AUC of 0.89 was achieved by multi-ROI-based maximal APTw value. Though suitable for application in clinical practice, the hot-spot ROI analysis might limit the reproducibility of the results. (3) different order feature and statistical methodology; conventional univariate analysis based on first-order features (maximum, minimum and other histogram features) were used in their study. In our study, apart from histogram features, higher order features were extracted. Moreover, radiomics analyses were performed for feature selection and classifier modeling.

Our study expands the current scarce but promising evidence on the diagnostic ability of APT_W_ images to predict *IDH1* mutation. Both univariate and radiomics analyses were performed. The efficacy achieved by radiomics analysis was superior to that with univariate analysis. The promising results may be partially attributed to two reasons: (1) Compared to first-order texture features based on histograms of the original image, higher order features provided spatial information among pixels and may better reflect the tumor heterogeneity (Hu et al., 2015); (2) Machine learning method enabled the integration of quantitative textural image features to build a model to predict *IDH1* mutation. Especially, the SVM classifier is considered to be a robust and effective machine-learning approach that has been predominately used in the fields of neuroimaging and molecular biology.

In addition, the promising prediction performance partly benefited from the feature selection procedure (to decrease the redundancy between features and the risk of model overfitting) by identifying a set of the most contributing features. Here, in the optimal feature subset for *IDH1* mutation prediction, most of the selected features were GLCM and GLRLM, which partly benefited from their high proportion in raw feature sets. However, it is worth noting that selected GLCM and GLRLM represent voxel-based change of grayscale and could reflect the complexity and heterogeneity of the tumor. Although the underlying biological mechanism for how these features relate to *IDH1* mutation status is presently unclear, our findings is in accordance with previous studies (Choi et al., 2016; Liu et al., 2018; Zhang et al., 2018). Besides, the results suggested that the feature subset does not necessarily require that each feature be very powerful, but the complementarity between features can help the subset achieve the best results.

There are several limitations in this study. First, our sample size was relatively small, especially for the *IDH1* wild-type group due to the inherent *IDH1* mutation distribution ratio in the general population. The results presented here require confirmation in a larger study. Second, only two-dimensional ROIs at each representative slice were delineated for feature extraction. Third, we did not thoroughly reveal the biological process behind the selected texture feature. Lastly, multi-model MRI data should be integrated into our model to improve efficiency of *IDH1* mutation prediction.

## Conclusion

In conclusion, the current study revealed that radiomic features derived from APT_W_ images are associated with *IDH1* mutation status in grade II/III gliomas. Using texture analysis and SVM, a machine learning model was established and the *IDH1* mutation status was predicted effectively. Our findings indicate that quantitative radiomics analysis based on APT_W_ images can potentially provide a non-invasive methodology for mutation status detection.

## Data Availability Statement

The datasets generated for this study are available on request to the corresponding author.

## Ethics Statement

The studies involving human participants were reviewed and approved by the Ethics Committee of Tangdu Hospital. Written informed consent to participate in this study was provided by the participants’ legal guardian/next of kin.

## Author Contributions

G-BC, L-FY, YH, and WW contributed to the conception and design of the study and drafting the work. YY, GX, Y-ZS, QT, and JZ contributed to the acquisition, analysis and interpretation of the data, and drafting and revision the work. All authors gave the final approval and agreement to all the aspects of the work.

## Conflict of Interest

The authors declare that the research was conducted in the absence of any commercial or financial relationships that could be construed as a potential conflict of interest.

## Footnotes

1 https://www.clinicaltrials.gov/

2 http://itk-snap.org
