# Supplementary material for: Amide Proton Transfer Imaging in Predicting Isocitrate Dehydrogenase 1 Mutation Status of Grade II/III Gliomas Based on Support Vector Machine
Source: Front Neurosci. 2020 Feb 21;14:144. doi: 10.3389/fnins.2020.00144 (PMC7047712; doi:10.3389/fnins.2020.00144)
Supplement: Supplementary file 1 [file Table_1.DOCX]

# Appendix 1

**Group1: First-Order Histogram Features
1. Minimum Intensity:** The value of the voxel(s) in the image ROI with the least value.

**2.** **Maximum Intensity:** The value of the voxel(s) in the image ROI with the greatest value.

**3. Median Intensity:** The median of the intensity or parameter values within the image ROI.

**4. Mean Intensity:** The mean of the intensity or parameter values within the image ROI.

$mean=\frac{1}{N}\sum_{i}^{N} X(i)$

**5. Standard Deviation:** Measures the amount of variation or dispersion from the mean of the values in the image ROI.

$s\tan dard deviation=\left(\frac{1}{N-1}\sum_{i=1}^{N} (X(i)-\bar{X})^{2}\right)^{1/2}$

**6.** **Variance:** The mean of the squared distances of each value in the image ROI from the mean of the values. This is a measure of the spread of the distribution about the mean.

$\mathrm{var}iance=\frac{1}{N-1}\sum_{i=1}^{N} (X(i)-\bar{X})^{2}$

**7. Voxel Count:** The total number of voxels within the ROI of the grayscale image or parameter map. Describe the size of the ROI.

**8.** **Voxel Value Sum:** The sum of voxels within the ROI of the grayscale image or parameter map.

**9.** **Range:** The difference between the highest and lowest voxel values within the image ROI.

**10.** **Root Mean Square:** The square-root of the mean of the squares of the values in the image ROI. It is another measure of the magnitude of the image values.

$RMS=\sqrt{\frac{\sum_{i}^{N}X(i)^{2}}{N}}$

**11.** **Mean Deviation:** The mean of the distances of each image value from the mean of all the values in the image ROI.

**12. Relative Deviation:** Let $\bar{X}$denote the mean of a set of quantities$X_{i}$, then the relative deviation is defined by:

$\frac{\Delta X_{i}}{\bar{X}}=\frac{|X_{i}-\bar{X}|}{\bar{X}}$

**13.** **Skewness:** Measures the asymmetry of the distribution of values in the image ROI about the mean of the values. Depending on where the tail is elongated and the mass of the distribution is concentrated, this value can be positive or negative.

$skewness=\frac{\frac{1}{N}\sum_{i=1}^{N}(X(i)-\bar{X})^{2}}{\left(\sqrt{\frac{1}{N}\sum_{i=1}^{N}(X(i)-\bar{X})^{2}}\right)^{2}}$

**14. Kurtosis:** A measure of the 'peakedness' of the distribution of values in the image ROI. A higher kurtosis implies that the mass of the distribution is concentrated towards the tail(s) rather than towards the mean. A lower kurtosis implies the reverse, that the mass of the distribution is concentrated towards a spike the mean.

$kurtosis=\frac{\frac{1}{N}\sum_{i=1}^{N}(X(i)-\bar{X})^{4}}{\left(\sqrt{\frac{1}{N}\sum_{i=1}^{N}(X(i)-\bar{X})^{2}}\right)^{2}}$

**15. Uniformity:** A measure of the sum of the squares of each discrete value in the image ROI. This is a measure of the heterogeneity of an image, where a lower uniformity implies a greater heterogeneity or a greater range of discrete image values.

$uniformity=\sum_{i=1}^{N_{l}} P(i)^{2}$

**16. Energy:** A measure of the magnitude of values in an image. A greater amount larger value implies a greater sum of the squares of these values.

$energy=\sum_{i}^{N} X(i)^{2}$

**17. Entropy:** Specifies the uncertainty in the image values. It measures the average amount of information required to encode the image values.

$entropy=-\sum_{i=1}^{N_{l}} P(i)\log_{2}P(i)$

**18. Frequency Size**

**19-37. Percentiles：**A percentile is a measure used in statistics indicating the value below which a given percentage of observations in a group of observations fall. There was 19 percentiles from Percentile5 to Percentile95 with the interval of 5.

**38-42. Quantiles：**For a finite population of *N* equally probable values indexed 1, …, $N$ from lowest to highest, the $k$-th $q$-quantile of this population can equivalently be computed via the value of:$\mathbf{Ip}=\text{ }\mathbf{N}\text{ }\mathbf{k}/\mathbf{q}$**.** Here, we have 5 quantiles, including Quantile0.025, Quantile0.25, Quantile0.5, Quantile0.75, Quantile0.975.

**Group2: Second-Order Texture Features**

While first order statistics describe the distribution of the gray values within an image, they do not contain information about the texture of a given region. This can be done by using gray level co-occurrence matrix (GLCM), gray level run length matrix (GLRLM) features, and gray level size zone matrix (GLSZM) features.

**2.1 Gray Level Co-occurrence Matrix (GLCM)**

The Gray level co-occurrence matrix (GLCM) $\Pr(i,j|\theta,d)$represents the joint probability of certain sets of pixels having certain grey-level values. It calculates how many times a pixel with grey-level **i** occurs jointly with another pixel having a grey value **j**. By varying the displacement vector **d** between each pair of pixels, we have 54 parameters related to each of the following 8 GLCM features.

**43-96.** **Energy of GLCM:** Also known as the Angular Second Moment and is a measure of the homogeneity of an image. A homogeneous image will contain less discrete gray levels, producing a GLCM with fewer but relatively greater values of P(i,j), and a greater sum of the squares.

$\sum_{i,j}^{(Formula)} g(i,j)^{2}$

$^{*}g$is a GLCM

Where $i,j$ are the spatial coordinates of$(i,j)$

**97-150. Entropy of GLCM:** Indicates the uncertainty of the GLCM. It measures the average amount of information required to encode the image values.

$-\sum_{i,j}^{Formula} g(i,j)\log_{2}(i,j)$

**151-204. Inertia of GLCM:** It reflects the clarity of the image and texture groove depth. The contrast is proportional to the texture groove, high values of the groove produces more clarity, in contrast small values of the groove will result in small contrast and fuzzy image.

$\sum_{i,j}^{Formula} ((i,j)^{2}\mathrm{g}(i,j))$

**205-258. Correlation:** Image-based Correlation measures the similarity of the grey levels in neighboring pixels, tells how correlated a pixel is to its neighbor over the whole image. Range = [-1 1]. Correlation is 1 or -1 for a perfectly positively or negatively correlated image.

$-\sum_{i,j}^{Formula} \frac{(i-\mu)(j-\mu)g(i,j)}{\sigma^{2}}$

**259-312. Inverse Difference Moment (IDM):** IDM is the local homogeneity. It is high when local gray level is uniform and inverse GLCM is high. IDM weight value is the inverse of the Contrast weight.

$\mathop{\sum\sum}\limits^{Formula} \frac{1}{1+(i-j)^{2}}g(i,j)$

**313-366. Cluster Shade:** A measure of the skewness and uniformity of the GLCM. A higher cluster shade implies greater asymmetry.

**367-420.** **Cluster Prominence:** A measure of the skewness and asymmetry of the GLCM. A higher value implies more asymmetry about the mean value while a lower value indicates a peak around the mean value and less variation about the mean.

$\sum_{i,j}^{Formula} ((i-\mu)+(j-\mu))^{4}g(i,j)$

**421-474.** **Haralick Correlation:** Measures the degree of similarity of the gray level of the image in the row or column direction. Represents the local grey level correlation, the greater its value, the greater the correlation;

$-\sum_{i,j}^{Formula} \frac{(i,j)g(i,j)-\mu_{t}^{2}}{\sigma_{t}^{2}}$

* where μ*_t_* and σ*_t_* are the mean and standard deviation of the row (or column, due to symmetry) sums.

**2.2 Gray Level Run Length Matrix (GLRLM)**

The gray level run length matrix (RLM) **(i, j** **|θ)** is defined as the numbers of runs with pixels of gray level *i* and run length *j* for a given direction θ. RLMs is generated for each sample image segment having directions (0°, 45°, 90° & 135°), then ten statistical features were derived, with 18 parameters related to each of these GLRLM features, similar to GLCM.

**475-528. Short Run Emphasis (SRE):** A measure of the distribution of short run lengths, with a greater value indicative of shorter run lengths and more fine textural textures.

$SRE(\theta)=\frac{1}{n_{r}}\sum_{i=1}^{M} \sum_{j=1}^{N} \frac{p(i,j,\theta)}{j^{2}}$

**529-582. Long Run Emphasis (LRE):** A measure of the distribution of long run lengths, with a greater value indicative of longer run lengths and coarser structural textures.

$LRE(\theta)=\frac{1}{n_{r}}\sum_{i=1}^{M} \sum_{j=1}^{N} p(i,j,\theta)j^{2}$

**583-636. Gray Level Non-uniformity (GLN):** Measures the similarity of gray level intensity values in the image, where a lower GLN value correlates with a greater similarity in intensity values.

$$GLN(\theta)=\frac{1}{n_{r}}\sum_{i=1}^{M} (\sum_{j=1}^{N} p(i,j,\theta))^{2}$$

**637-690. Run Length Non-uniformity (RLN):** Measures the similarity of run lengths throughout the image, with a lower value indicating more homogeneity among run lengths in the image.

$RLN(\theta)=\frac{1}{n_{r}}\sum_{j=i}^{N} (\sum_{i=1}^{M} p(i,j,\theta))^{2}$

**691-744. Low Gray Level Run Emphasis (LGRE):** Measures the distribution of low gray-level values, with a higher value indicating a greater concentration of low gray level values in the image.

$LGRE(\theta)=\frac{1}{n}\sum_{j=i}^{N} \sum_{i=1}^{M} \frac{p(i,j,\theta)}{i^{2}}$

**745-798. High Gray Level Run Emphasis (HGRE):** Measures the distribution of the higher gray-level values, with a higher value indicating a greater concentration of high gray level values in the image.

$HGRE(\theta)=\frac{1}{n_{r}}\sum_{j=i}^{N} \sum_{i=1}^{M} p(i,j,\theta)i^{2}$

**305-852. Short Run Low Gray Level Emphasis (SRLGE):** Measures the joint distribution of shorter run lengths with lower gray level values.

$SRLGE(\theta)=\frac{1}{n_{r}}\sum_{j=i}^{N} \sum_{i=1}^{M} \frac{p(i,j,\theta)}{i^{2}j^{2}}$

**853-906. Short Run High Gray Level Emphasis (SRHGE):** Measures the joint distribution of shorter run lengths with higher gray level values.

$SRHGE(\theta)=\frac{1}{n_{r}}\sum_{j=i}^{N} \sum_{i=1}^{M} \frac{p(i,j,\theta)t^{2}}{j^{2}}$

**907-960. Long Run Low Gray Level Emphasis (LRLGE):** Measures the joint distribution of long run lengths with lower gray level values.

$LRLGE(\theta)=\frac{1}{n_{r}}\sum_{j=i}^{N} \sum_{i=1}^{M} \frac{p(i,j,\theta)j^{2}}{t^{2}}$

**961-1014. Long Run High Gray Level Emphasis (LRHGE):** Measures the joint distribution of long run lengths with higher gray level values.

$LRHGE(\theta)=\frac{1}{n_{r}}\sum_{j=i}^{N} \sum_{i=1}^{M} p(i,j,\theta)i^{2}j^{2}$

**2.3 Gray Level Size Zone Matrix (GLSZM)**

GLSZM is the starting point of Thibault matrices. For a texture image f with N gray levels, it is denoted GSf(s, g) and provides a statistical representation by the estimation of a bivariate conditional probability density function of the image distribution values. It is calculated according to the pioneering Run Length Matrix principle: the value of the matrix GSf(s, g) is equal to the number of zones of size s and of gray level g. The resulting matrix has a fixed number of lines equal to N, the number of gray levels, and a dynamic number of columns, determined by the size of the largest zone as well as the size quantization.

**1015. Small Zone Emphasis**

$SZE=\sum_{i} \sum_{j} \frac{p(i,j)}{j^{2}}$

**1016. Large Zone Emphasis**

$LZE=\sum_{i} \sum_{j} j^{2}p(i,j)$

**1017. Zone Percentage**

$ZP=\sum_{i} \sum_{j} \frac{\sum_{i}\sum_{j}p(i,j)}{\sum_{j}j\sum_{i}p(i,j)}$

**1018. Low Gray Level Zone Emphasis**

$LGZE=\sum_{i} \sum_{j} \frac{p(i,j)}{i^{2}}$

**1019. High Gray Level Zone Emphasis**

$HGZE=\sum_{i} \sum_{j} i^{2}p(i,j)$

**1020. Small Zone Low Gray Level Emphasis**

$SZLGE=\sum_{i} \sum_{j} \frac{p(i,j)}{i^{2}j^{2}}$

**1021. Small Zone High Gray Level Emphasis**

$SZLGE=\sum_{i} \sum_{j} \frac{j^{2}p(i,j)}{i^{2}}$

**1022. Large Zone Low Gray Level Emphasis**

$LZLGE=\sum_{i} \sum_{j} \frac{j^{2}p(i,j)}{i^{2}}$

**1023. Large Zone High Gray Level Emphasis**

$LZHGE=\sum_{i} \sum_{j} i^{2}j^{2}p(i,j)$

**1024. Gray Level Variance**

$GLV=\frac{1}{Ng\times Lz}\sum_{i} \sum_{j} (ip(i,j)-\mu_{i})^{2}$

**1025. Zone-Size Variance**

$ZSV=\frac{1}{Ng\times Lz}\sum_{i} \sum_{j} (jp(i,j)-\mu_{j})^{2}$

**Group3: Form Factor Parameters**This group of features includes descriptors of the three-dimensional size and shape of the tumor region. Let in the following definitions ***V*** denote the volume and ***A*** the surface area of the volume of interest. We determined the following shape and size based features:

**1026. Sphericity**

$sphericity=\frac{\pi^{\frac{1}{3}}(6V)^{\frac{2}{3}}}{A}$

**1027. Surface area**

$A=\sum_{i=1}^{N} \frac{1}{2}|a_{i}b_{i}\times a_{i}c_{i}|$

**1028. Compactness 1**

$compactness 1=\frac{V}{\sqrt{\pi}A^{\frac{2}{3}}}$

**1029. Compactness 2**

$compactness 2=36\pi\frac{V^{2}}{A^{3}}$

**1030. Maximum 3D diameter**

**1031. Spherical disproportion**

$spherical disproportion=\frac{A}{4\pi R^{2}}$

**1032. Surface to volume ratio**

$surface to volume ratio =\frac{A}{V}$

**1033-1034. VolumeCC and VolumeMM**

**Group4: Haralick Parameters**

**1035. Angular Second Moment (ASM)**

$f_{1}=\sum_{i=1}^{N_{g}} \sum_{j=1}^{N_{g}} \left(\frac{P(i,j)}{R}\right)^{2}=\sum_{i} \sum_{j} p(i,j)^{2}$

**1036. Contrast:** The contrast feature is a difference moment of the P matrix and is a measure of the contrast or the amount of local variations present in the image.

$f_{2}=\sum_{k=0}^{N_{g}-1} k^{2}\left\{\sum_{i=1}^{N_{g}} \sum_{j=1}^{N_{g}} \delta_{\left|i-j\right|,k}p(i,j)\right\}=\sum_{k=0}^{N_{g}-1} k^{2}p_{x-y}(k)$

**1037. HaraVariance**

$f_{4}=\sum_{i=1}^{N_{g}} \sum_{j=1}^{N_{g}} (i-\mu)^{2}p(i,j)$

**1038. Inverse Difference Moment**

$f_{5}=\sum_{i=1}^{N_{g}} \sum_{j=1}^{N_{g}} \frac{1}{1+(i-j)^{2}}p(i,j)$

**1039. Sum Average**

$f_{6}=\sum_{i=2}^{2N_{g}} ip_{x+y}(i)$

**1040. Sum Variance**

$f_{7}=\sum_{i=2}^{2N_{g}} (i-f_{8})^{2}p_{x+y}(i)$

**1041. Sum Entropy**

$f_{8}=-\sum_{i=2}^{2N_{g}} p_{x+y}(i)\log(p_{x+y}(i))$

**1042. Haralick Entropy**

$f_{9}=-\sum_{i=1}^{N_{g}} \sum_{j=1}^{N_{g}} p(i,j)\log(p(i,j))$

**1043. Difference Variance**

$f_{10}=variance of p_{x-y}$

**1044. Difference Entropy**

$f_{11}=-\sum_{i=0}^{N_{g}-1} p_{x-y}(i)\log(p_{x-y}(i))$
